# Decision-grade digital twins for *in situ* bioremediation of contaminated soil and groundwater: a critical review

**DOI:** 10.3389/fbioe.2026.1866957

**Published:** 2026-07-01

**Authors:** Mariusz Cycoń

**Affiliations:** Department of Microbiology, Faculty of Pharmaceutical Sciences, Medical University of Silesia, Sosnowiec, Poland

**Keywords:** adaptive decision support, contaminated soil and groundwater, decision-grade digital twins, *in situ* bioremediation, microbial process monitoring, multiple lines of evidence

## Abstract

Field claims about *in situ* bioremediation often fail because monitoring data are not mapped to the causal process they are intended to prove. This critical review defines decision-grade digital twins as inferential systems, not as visualization tools, data lakes, or calibrated trend models. A decision-grade twin must connect a living conceptual site model with evidence streams that test competing hypotheses, prediction models with stated admissibility limits, uncertainty updates, and auditable trigger logic. The review evaluates how geochemistry, compound-specific isotope analysis, functional genes and transcripts, flux measurements, geophysical imaging, toxicity endpoints, and high-frequency sensing can support claims about pathway activation, treatment limitation, rebound risk, secondary impacts, and closure readiness. It also distinguishes the decision value of reactive transport models, hybrid process-data models, surrogate models, data-driven surveillance, and microbial process representations. Across hydrocarbons and polycyclic aromatic hydrocarbons, chlorinated solvents, pesticides, pharmaceuticals, and per- and polyfluoroalkyl substances, decision value depends on whether the evidence can distinguish among reaction, retention, redistribution, metabolite formation, and durable risk reduction. The review concludes that digital twins deserve decision-grade status only when they reduce ambiguity that can affect intervention choice, operating intensity, switching criteria, monitoring design, secondary-risk management, or closure judgment.

## 1 Introduction

*In situ* bioremediation of contaminated soil and groundwater is still judged too often by one signal that is easy to measure and dangerously easy to misread, i.e., concentration decline. In heterogeneous subsurface systems, lower dissolved concentrations may reflect dilution, dispersion, boundary-condition shifts, transient redox change, or diffusion-limited release from low-permeability domains rather than biologically mediated contaminant destruction (Brennan et al., 2006; Hunkeler et al., 2011; Majone et al., 2015; O’Connor et al., 2018; Romantschuk et al., 2023). This problem is not semantic. It determines whether a site is interpreted as progressing toward closure or merely redistributing unresolved mass. The central inferential task in field bioremediation is therefore not to document change, but to decide what kind of change has occurred and whether microbial activity is truly responsible.

That inferential task has become more difficult, not less, as monitoring systems have expanded. Contaminated sites now generate far more data than most management frameworks can interpret causally. Routine chemistry, geochemical indicators, isotope signatures, functional genes, high-frequency redox measurements, geophysical data, and flux metrics do not constrain the same part of the system, do not respond on the same spatial or temporal scale, and do not carry equal decision value (Song et al., 2017; Horst et al., 2022; Huang et al., 2022; Davis et al., 2023; Irianni-Renno et al., 2024; Nivorlis et al., 2024). Yet the literature still tends to treat data accumulation as if it were equivalent to interpretive progress. It is not. A site with many poorly aligned indicators is not better understood than a site with fewer measurements chosen to test competing hypotheses. The bottleneck in current practice is not only weak linkage between observations, mechanism, prediction, and action. It is also the scarcity of observations with sufficient spatial support, temporal density, quality assurance (QA)/quality control (QC) integrity, and mechanism specificity to close a decision. A twin therefore becomes decision-grade only for the decision it can actually support; for many sites the defensible output will remain a bounded surveillance system, not a control-ready inferential system.

This weakness is especially consequential when remediation depends on microbial processes whose field expression is contingent on redox state, amendment delivery, source architecture, electron-acceptor competition, and mass-transfer constraints. The same intervention can produce sharply different outcomes depending on whether the limiting step is pathway activation, donor access, metabolite turnover, or back diffusion from a layer with low permeability (Rahm and Richardson, 2008; Scheutz et al., 2008; Hunkeler et al., 2011; Chambon et al., 2013; Ottosen et al., 2021; De Vera et al., 2022). This is evident across chlorinated solvents, petroleum hydrocarbons and polycyclic aromatic hydrocarbons (PAHs), pesticides, pharmaceuticals, and per- and polyfluoroalkyl substances (PFASs), where apparent treatment response may diverge from actual pathway closure, metabolite acceptability, or long-term risk reduction (Tortella et al., 2012; Song et al., 2017; Cycoń et al., 2019; Davis et al., 2023; Romantschuk et al., 2023; Klim et al., 2025). Microbial evidence is thus indispensable, but it is rarely self-sufficient. Outside process context, biological signals can indicate capacity without activity, presence without control, or transformation without acceptable outcome. This distinction is now central to molecular monitoring in soil bioremediation: detection of genes or community shifts can support pathway potential, but verified pathway operation requires activity-linked evidence, product formation, and consistency with geochemical and transport constraints (Cycoń, 2026).

Digital twins have entered this landscape with promise but also terminological drift. Outside remediation, the term often denotes updated virtual representations, model-data systems, or operational decision-support architectures (Bécue et al., 2020; Lim et al., 2020; Rasheed et al., 2020; Singh et al., 2021; Schneider et al., 2022; Emmert-Streib, 2023; Dihan et al., 2024; Durden, 2025; Underhill et al., 2026). For contaminated sites, this general definition is insufficient unless the system also states which conceptual site model (CSM) alternatives are being tested, which observations constrain which states, and what decision can change if the inference changes. The same overstatement appears in modeling. Reactive transport models (RTMs), surrogate models, hybrid architectures, and microbial process models can each support field interpretation, but only within the evidentiary limits that make their claims admissible. If concentration trends are fitted without independent constraints on transport, redox regime, pathway activity, or mass exchange, good agreement with data may simply mask structural error (Adamson et al., 2016; Refsgaard et al., 2016; Aliashrafi et al., 2021; Schneider et al., 2022; Verhaeghe et al., 2024). High-frequency sensing does not solve this problem by itself. It can sharpen state estimation and reveal regime switching, yet it can also exacerbate overfitting, propagate sensor artifacts, and create false confidence if sequential updating is not disciplined by process understanding and quality control (Sale et al., 2021; Horst et al., 2022; Huang et al., 2022; Davis et al., 2023). The critical question is therefore not whether digital twins or advanced models can be built, but which decision claims they can defend under conditions of field heterogeneity. Current practice still treats uncertainty as something that can be reduced after monitoring and modeling are assembled. In contaminated subsurface systems, the opposite is true. Competing site hypotheses, pathway non-closure, low-permeability mass exchange, and secondary impacts are not residual complications, but determinants of what can be inferred at all (Wanner et al., 2016; Song et al., 2017; Haasnoot et al., 2018; O’Connor et al., 2018; Davis et al., 2023; Verhaeghe et al., 2024).

This article is a critical review rather than a systematic review or meta-analysis. The purpose is not to estimate effect sizes or exhaustively enumerate all digital platforms, but to determine which combinations of microbial evidence, geochemical context, transport information, and prediction structure can support defensible field judgments *in situ* bioremediation. Therefore, the literature was coded using five attributes, i.e., contaminant class, decision type, dominant uncertainty, principal lines of evidence, and the decision claim that the study could support. Field studies were weighted most strongly when they linked observations to a named management decision or to a falsifiable site hypothesis. Modeling papers were weighted when the model claim was bounded by independent evidence, explicit uncertainty, or validation outside calibration. General digital-twin papers were used only to define architecture, updating, data governance, or model-data coupling, not to infer remediation performance. Searches were conducted in Web of Science, Scopus, PubMed, Google Scholar, ScienceDirect, ACS Publications, SpringerLink, Wiley, MDPI, and publisher databases using combinations of the following terms: digital twin, environmental digital twin, remediation, *in situ* bioremediation, bioaugmentation, reactive transport model (RTM), contaminated groundwater, contaminated soil, chlorinated ethenes, polycyclic aromatic hydrocarbons (PAHs), pesticides, pharmaceuticals, per- and polyfluoroalkyl substances (PFASs), compound-specific isotope analysis (CSIA), functional genes, quantitative PCR (qPCR), quantitative reverse transcription PCR (qRT-PCR), transcripts, mass flux, redox monitoring, geophysical imaging, uncertainty, identifiability, and decision support. Studies were included when they addressed at least one of the following: causal attribution of degradation, model admissibility for a field decision, evidence integration under heterogeneity, uncertainty that can alter remediation action, or governance of a digital-twin workflow. Studies were excluded when “digital twin” meant only visualization, generic data aggregation, or prediction without a stated decision claim. The representative-study counts in Table 1 refer to papers explicitly used in this review to support each domain, not to the total number of publications retrieved by the search.

**TABLE 1:** Scope and evidence-weighting matrix used for the critical review.

Domain	Main decision types	Dominant uncertainty	Primary lines of evidence	Study counts
Hydrocarbons and PAHs	Intensify aeration, adjust nutrient supply, evaluate post-oxidation biostimulation, judge flux reduction	Bioavailability, mass transfer, volatilization *versus* biodegradation, residual toxicity	TPH or PAH trends, respiration, oxygen demand, flux, toxicity, rebound after stimulation	8
Chlorinated solvents	Adjust donor, bioaugment, revise delivery, switch technology, assess closure risk	Pathway completion, donor competition, back diffusion, daughter-product accumulation	Parent-daughter products, ethene, redox, CSIA, *vcrA* and *tceA*, flux, mass-transfer diagnostics	12
Pesticides and pharmaceuticals	Distinguish removal from detoxification, revise redox or bioaugmentation strategy, block closure when metabolites persist	Partial transformation, metabolite hazard, sorption-retention trade-offs, redox-sensitive pathways	Parent and metabolite profiles, toxicity, redox, functional genes or transcripts, pathway-specific chemistry	13
PFASs	Separate retention from destruction, evaluate precursor transformation, judge mobility control, avoid premature closure	Sorption, electrostatic partitioning, air-water interfacial retention, precursor conversion, incomplete defluorination, product toxicity	Precursor and product profiles, total oxidizable precursor assay where applicable, extractable and adsorbed mass, fluoride release, total fluorine or organofluorine balance, mobility and toxicity endpoints	5
Digital-twin architecture and governance	Decide whether a reported system is a digital representation, operational support tool, or decision-grade twin	Terminological drift, update opacity, data provenance, weak decision trace	CSM ontology, observation-to-state map, update gates, versioning, data pipeline, trigger records	17

CSIA, compound-specific isotope analysis; CSM, conceptual site model; PAHs, polycyclic aromatic hydrocarbons; PFASs, per- and polyfluoroalkyl substances; *tceA*, trichloroethene reductive dehalogenase gene; TPH, total petroleum hydrocarbons; *vcrA*, vinyl chloride reductive dehalogenase gene. Counts are not prevalence estimates. They identify the representative papers explicitly used to support each part of the argument.

## 2 Why most remediation digital twins are not decision-grade

The current literature uses the term digital twin too loosely for contaminated-site remediation. In many cases, the label is assigned to systems that aggregate measurements, display site status, or run scenario forecasts, yet do not specify which competing site hypotheses are being tested, which observations constrain which state variables, or how uncertainty is propagated into a field decision. That usage is not merely imprecise. It obscures the main distinction that matters in practice, i.e., the difference between a digital representation of a contaminated site and an inferential framework capable of changing remediation action under uncertainty. The central error in the present paradigm is therefore categorical. The field often treats digitalization as evidence of decision maturity, even when the underlying system has not advanced beyond descriptive data management.

**FIGURE 1:**
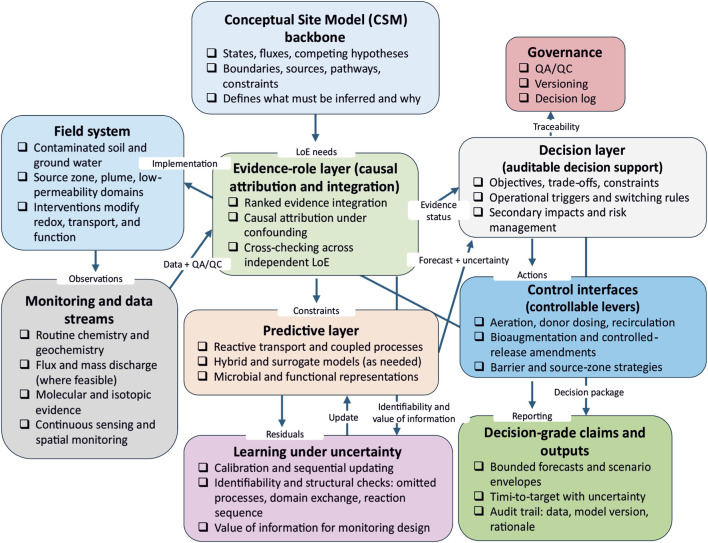
Minimum causal architecture of a decision-grade digital twin for *in situ* bioremediation. Observations enter the CSM, evidence, prediction, uncertainty, and decision layers only through traceable QA/QC and update rules. CSM, conceptual site model; LoE, line of evidence; QA, quality assurance; QC, quality control.

**FIGURE 2:**
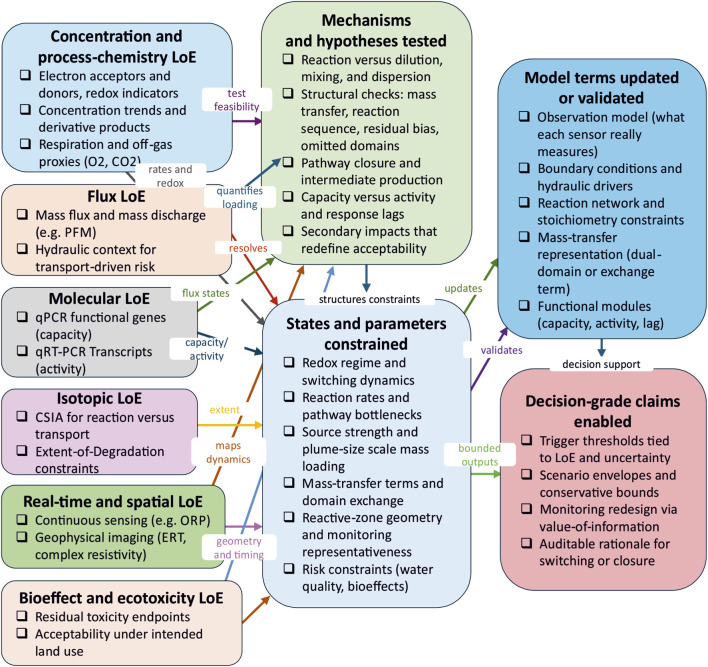
Evidence-to-inference map for decision-grade claims. The figure shows how evidence streams constrain site states, model terms, structural checks, and action thresholds. Here, structural checks are tests for omitted process domains, wrong pathway sequence, residual bias, or invalid boundary conditions, rather than additional calibration. CSIA, compound-specific isotope analysis; ERT, electrical resistivity tomography; LoE, line of evidence; ORP, oxidation-reduction potential; PFM, passive flux meter; qPCR, quantitative PCR; qRT-PCR, quantitative reverse transcription PCR.

This problem becomes obvious once the conceptual site model (CSM) is treated as more than a static narrative. In remediation, the CSM must encode source architecture, contaminant phases, reactive zones, transport barriers, redox structure, and the causal links between microbial activity and contaminant transformation. These elements define the state variables to be estimated, the observations that can constrain them, and the alternative hypotheses that must remain explicit during model updating. Competing hypotheses about source control, back diffusion, pathway limitation, or redox switching imply different intervention logic and different expectations for persistence, rebound, or apparent progress under matrix diffusion. A remediation twin that does not formalize such alternatives is not testing the site; it is only narrating it. Studies on chlorinated solvents, BTEX (benzene, toluene, ethylbenzene, and xylene) systems, isotope-based source discrimination, and continuous redox monitoring reach this conclusion from different directions: unless the site model is expressed as a causal structure with observable consequences, interpretation reverts to expert judgment presented as data science (Kao et al., 2008; Blessing et al., 2009; Hunkeler et al., 2011; Lim et al., 2020; Sale et al., 2021; De Vera et al., 2022; Schneider et al., 2022; Verhaeghe et al., 2024). The literature is already sufficient on this point. The unresolved issue is not whether CSMs matter, but why so many remediation workflows still fail to encode them in a form that can discipline inference. Figure 1 operationalizes these criteria by showing the minimum inferential architecture required for a remediation twin to support auditable field decisions. In Figures 1, 2, structural checks means explicit tests of whether the assumed process structure is wrong, not additional parameter tuning. Examples include omitted source domains, absent matrix-diffusion terms, incorrect reaction sequence, misassigned redox windows, boundary-condition error, and residuals that cluster by well, depth, event, or time period.

Maturity frameworks developed for wastewater treatment, drainage, water-distribution, and broader water-sector applications clarify why sensor-model coupling, state estimation, model updating, and operational support matter (Bartos and Kerkez, 2021; Pedersen et al., 2021; Torfs et al., 2022; Wang et al., 2024; Ghorbani Bam et al., 2025; Hartmann et al., 2025; Singh et al., 2025). Their criteria cannot be transferred directly to contaminated aquifers. In a water-distribution network, pressure, flow, valve status, and boundary conditions are often observed or controlled at operationally relevant scales, and digital-twin implementations can therefore be evaluated against comparatively explicit hydraulic and operational states (Conejos Fuertes et al., 2020). In an aquifer undergoing bioremediation, the decision-critical states are partly hidden: source architecture, reactive-zone contact, low-permeability exchange, redox microzonation, pathway activity, and product acceptability. A forecast that is operationally adequate in a pipe network may therefore remain inadmissible for closure or remedy switching at a contaminated site. Table 2 summarizes this domain mismatch by contrasting controllability, observability, calibration targets, and the consequences of erroneous decisions in engineered water systems and contaminated-aquifer remediation.

**TABLE 2:** Why water-system digital-twin maturity criteria do not suffice for contaminated-aquifer remediation.

Dimension	Water and wastewater systems	*In situ* bioremediation of aquifers	Consequence for decision-grade claims
Controllability	Pumps, valves, aeration, and treatment units are commonly known control points	Donor, oxygen, amendments, or organisms may not reach the controlling mass fraction	Control action must be tied to delivery evidence
Observability	Pressure, flow, quality, and asset status may be monitored at high density	Source zones, redox interfaces, biomass activity, and matrix diffusion remain partly hidden	Monitoring must test CSM alternatives, not only trends
Calibration target	Short-term hydraulic or process response can often be checked against dense observations	Similar concentration fits may represent reaction, dilution, retention, or back diffusion	Validation must include independent lines of evidence
Cost of wrong action	Service quality, treatment efficiency, overflow, or asset operation may degrade	Closure, plume migration, metabolite accumulation, collateral water-quality damage, or futile intensification may result	Trigger rules require stronger causal attribution

CSM, conceptual site model. The comparison does not imply that water-sector digital-twin frameworks are technically weaker. It identifies the additional evidentiary burden created by partial observability, heterogeneous source architecture, matrix diffusion, redox microzonation, and mechanism-specific remediation endpoints in contaminated aquifers.

The contrast is not one of technological maturity but of evidentiary burden. Air-sparging studies, isotope work in aquitards, uranium bioremediation, and chlorinated-solvent field studies show that credibility depends on whether observations exclude plausible confounders, including transport, sorption, diffusion, redox displacement, and incomplete pathway closure (Wu et al., 2006; Hunkeler et al., 2011; Alessi et al., 2014; Gedalanga et al., 2014; Adamson et al., 2015; Wanner et al., 2016; O’Connor et al., 2018; Aliashrafi et al., 2021; Ottosen et al., 2021). A further weakness of the field is its tendency to confuse operational responsiveness with scientific admissibility. The promise of adaptive remediation is attractive, particularly where donor addition, recirculation, or aeration can be adjusted in response to incoming observations. Yet adaptive behavior is not intrinsically a mark of scientific quality. In a subsurface setting, a fast control loop can amplify the consequences of misclassification if the process state is inferred from ambiguous proxies. Continuous oxidation-reduction potential (ORP) measurements, for example, may reveal regime switching that periodic sampling misses, but ORP alone cannot establish contaminant-specific pathway completion, nor can it distinguish a productive redox shift from one that increases metabolite risk or collateral water-quality damage. The same caution applies to data-driven anomaly detection and hybrid emulators. Speed is useful, but speed without causal discipline is only a faster route to false confidence. The literature does not support a generalized claim that remediation twins improve decisions by becoming more adaptive. It supports the narrower claim that adaptation is justifiable only when the system has already solved the attribution problem for the signal on which it intends to act.

The admission test is claim-specific, not a generic maturity scale. Each criterion is scored as pass, conditional pass, or fail for a stated decision claim. A pass requires that the publication reports the evidence, mapping, validation, or governance item explicitly. A conditional pass is allowed only when the limitation is stated and the decision claim is narrowed accordingly. A fail on any necessary criterion prevents a decision-grade claim for that decision type. Table 3 operationalizes this claim-specific standard by separating necessary and supporting criteria for three decision types that carry different evidentiary burdens, i.e., intensification of an ongoing treatment, technology switching, and closure or reduction of monitoring. The same admission test can also be read as a stakeholder-risk hierarchy. Low-stakes operational adjustments, such as minor aeration, donor-pulse, recirculation, or sampling-frequency changes within a preapproved operating envelope, require evidence that the current state has shifted and that the proposed adjustment is reversible. Intermediate-stakes decisions, such as sustained intensification, delivery redesign, or bioaugmentation, require stronger attribution because they can create secondary water-quality effects, alter redox state, or consume limited site access. High-stakes decisions, such as technology switching, monitoring reduction, regulatory closure, or remedy termination, require the highest evidentiary burden because false confidence can leave residual mass, rebound potential, daughter products, or exposure pathways unresolved. A twin that is adequate for adjusting aeration or donor timing is therefore not automatically adequate for closure, because the stakeholder consequence of error is different.

**TABLE 3:** Claim-specific admission test for decision-grade remediation digital twins.

Criterion	Scoring test	Intensify current treatment	Switch technology	Close or reduce monitoring	Fail state
Competing CSMs	At least two plausible site hypotheses are stated with observable consequences	Necessary	Necessary	Necessary	A single preferred site story is treated as fact
Observation-to-state map	Key states, parameters, boundaries, and triggers are linked to named observations, with scale and uncertainty	Necessary	Necessary	Necessary	Measurements are pooled without saying what they constrain
Reaction *versus* transport separation	The claim is constrained by independent evidence such as CSIA, products, biomarkers, flux, mass balance, or mass-transfer diagnostics	Necessary for biodegradation-based intensification	Necessary	Necessary	Parent-compound decline is treated as treatment proof
Validation outside calibration	Forecasts are checked against holdout periods, independent wells, independent LoE, or intervention events	Supporting if action is low risk	Necessary	Necessary	Fit to historical data is the only validation
Identifiability and structural alternatives	Alternative parameter sets or model structures are tested, and unresolved ambiguity is retained in the decision record	Supporting, potentially necessary depending on risk	Necessary	Necessary	One calibrated model is treated as the site truth
Uncertainty envelope and stop conditions	Predictions include bounded scenarios, decision limits, and conditions that invalidate the current action	Necessary	Necessary	Necessary	Only best estimates are reported
Trigger and switching logic	The evidence required, action allowed, uncertainty tolerated, and approval pathway are stated before action	Necessary	Necessary	Supporting, potentially necessary depending on regulatory use	Actions follow informal interpretation
Versioned decision trace	Data version, model version, uncertainty statement, approval record, and action are linked	Supporting for one-time scientific interpretation	Necessary	Necessary	Learning cannot be reconstructed

CSIA, compound-specific isotope analysis; CSM, conceptual site model; LoE, line of evidence. Criteria are evaluated for the stated decision claim, not for the digital-twin system in the abstract. “Necessary” means that the criterion must be satisfied to support the listed decision type. “Supporting” means that the criterion strengthens the claim but is not sufficient by itself. A fail on any necessary criterion prevents a decision-grade claim for that decision type.

To avoid treating the admission test as merely a normative proposal, Table 4 applies it to representative publications and distinguishes full decision-grade claims from narrower contributions, including monitoring frameworks, model structures, spatial evidence layers, and feasibility demonstrations.

**TABLE 4:** Illustrative application of the admission test to representative publications.

Publication	Reported contribution	Main support for the admission test	Missing element for full decision-grade status
Sale et al. (2021)	Real-time spatial and temporal monitoring of biogeochemical potentials	High value for regime detection and update timing	Does not by itself prove contaminant-specific pathway completion or closure readiness
Sookhak Lari et al. (2022)	Digital-twin feasibility for NSZD site characterization	Links site data, source-zone behavior, and model interpretation	Decision trace and regulatory trigger logic remain limited
Adamson et al. (2016)	Air-sparging and mass-transfer limitation evidence	Strong constraint on tailing, rebound, and transport confounding	Not a digital-twin workflow with update governance
Luo et al. (2024)	Reactive transport modeling for chlorinated ethenes	Strong model-structure guidance for pathway and transport coupling	Not a field digital twin with audit and switching rules
Nivorlis et al. (2024)	Geophysical monitoring for remediation interpretation	Supports spatial constraint on delivery and reactive-zone geometry	Does not establish reaction mechanism without chemical or biological coupling
Schneider et al. (2022)	Hybrid modeling practice for water-resource recovery systems	Useful for model-data interfaces and hybrid model discipline	Not specific to subsurface contamination or bioremediation decisions
Verhaeghe et al. (2024)	Good modeling practice for parallel hybrid models	Strong support for hybrid-model validation and release discipline	Does not solve remediation-specific attribution
Davis et al. (2023)	Decision-linked monitoring and interpretation for contaminated sites	Supports uncertainty, monitoring design, and evidence integration	The admission test must still be applied to site-specific reports

NSZD, natural source zone depletion. The assessment is illustrative rather than exhaustive. It evaluates the decision-grade claim supported by each publication as reported, not the scientific quality or general importance of the study. A study may be highly valuable as a monitoring framework, model structure, spatial evidence layer, or feasibility demonstration without satisfying the full admission test for control-linked field decisions, remedy switching, monitoring closure, or regulatory closure.

## 3 Contaminant classes, microbial mechanisms, and the limits of generic control logic

**FIGURE 3:**
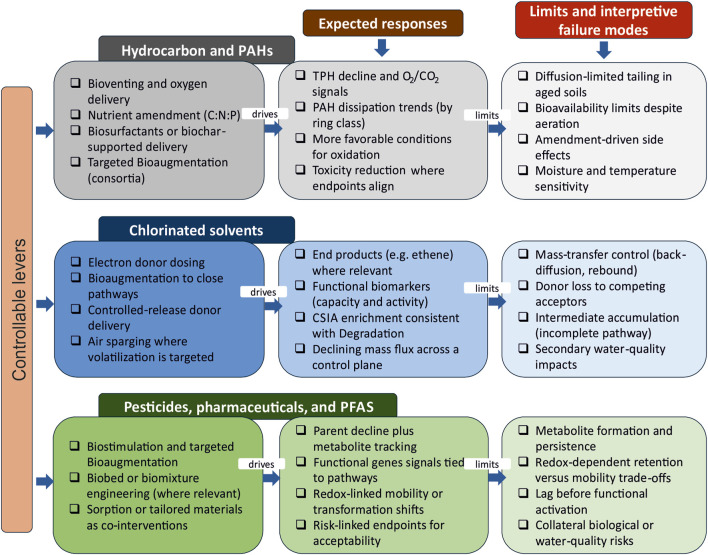
Contaminant-specific control levers and recurrent failure modes in situ bioremediation. The map indicates where a control action may fail because the dominant limitation differs by contaminant class. CSIA, compound-specific isotope analysis; PAHs, polycyclic aromatic hydrocarbons; PFASs, per- and polyfluoroalkyl substances; TPH, total petroleum hydrocarbons.

The next error in current remediation thinking is subtler, but just as damaging. The field often writes as if digital control logic can be abstracted from contaminant class and then applied across sites once the monitoring architecture is in place. That assumption fails because the limiting microbial process is not the same across hydrocarbon and PAH systems, chlorinated solvents, pesticides and pharmaceuticals, and PFASs. The same operational lever can therefore have different meanings, different failure modes, and different decision thresholds depending on which transformation pathway is intended and which constraint dominates in the field. A digital twin that does not begin from mechanism-specific control failure is not transferable. It is generic only in the weak sense that it is equally underdetermined everywhere. Figure 3 links operational levers to process-level expectations and to the points at which interpretation becomes unreliable. In this review, it serves to show that field decision logic must be contaminant-specific and mechanism-specific, rather than transferred across classes by analogy. Mechanism-specific failure is therefore treated here as the organizing unit of digital-twin logic. Hydrocarbon and PAH systems fail when delivery metrics are mistaken for access to residual mass; chlorinated-solvent systems fail when donor response is mistaken for complete dechlorination; pesticide and pharmaceutical systems fail when parent-compound loss is mistaken for detoxification; and PFAS-impacted systems fail when retention or precursor conversion is mistaken for destruction.

Hydrocarbon and PAH systems illustrate why simple enhancement logic is often overstated. Oxygen delivery, nutrient balance, and respiration monitoring are important, but field performance is often limited less by nominal amendment sufficiency than by accessibility of the contaminant fraction that remains. Vadose-zone bioventing studies, laboratory and mesocosm work on PAHs, and field observations on stratified response all point in the same direction, i.e., aeration can accelerate early removal without solving the controlling problem when contaminants are retained in microporous domains or when hydrophobicity restricts microbial access (Hamdi et al., 2007; Milic et al., 2009; Frutos et al., 2010; Silva-Castro et al., 2013; Dahan et al., 2017; Haleyur et al., 2019; Simpanen et al., 2016). The classic contrast between near-complete dissipation of anthracene or pyrene and the persistence of benzo [a]pyrene is not simply a matter of slower kinetics. It reveals a shift in the dominant bottleneck from metabolic competence to bioavailability and mass transfer. Chemical pre-oxidation followed by biostimulation may improve access to poorly available fractions, yet those sequences can also mobilize contaminant mass or create toxicity issues that invalidate concentration-based success claims. The synthesis is clear. In hydrocarbon and PAH settings, digital twins should not treat oxygen response as a surrogate for treatment sufficiency. They should ask whether the intervention is changing access to the controlling fraction of mass and whether flux, not only concentration, is moving in the right direction.

Chlorinated solvent remediation makes the inadequacy of generic control logic even clearer. Air sparging, enhanced reductive dechlorination (ERD), controlled-release donor systems, and electro-bioremediation do not target the same limiting process and cannot be judged with the same evidence threshold. Air sparging can reduce dissolved concentrations quickly, but laboratory and field evidence show persistent tailing when diffusion from poorly connected pores governs late-stage release (Adams et al., 2011; Adamson et al., 2016; O’Connor et al., 2018). ERD poses a different question: whether the system progresses through the reductive pathway to benign end products under donor limitation, electron-acceptor competition, and spatially variable redox conditions (Rahm and Richardson, 2008; Scheutz et al., 2008; Hunkeler et al., 2011; Chambon et al., 2013; Ottosen et al., 2021; Luo et al., 2024). Controlled-release donor systems and enhanced *in situ* bioremediation agents should be treated as delivery variables rather than formulation details, because distribution, residence time, release kinetics, and contact with the reactive zone determine whether the intended pathway can be sustained (Liang et al., 2013; O’Connor et al., 2018; Xie et al., 2024; Bhattacharya et al., 2025). Electro-bioremediation adds another control mode because imposed electrochemical gradients can alter electron transfer, redox zoning, and amendment distribution, but it still requires independent evidence that contaminant transformation, not only field perturbation, has occurred (Aulenta et al., 2025). A chlorinated-solvent twin must therefore test pathway completion, donor competition, and mass-transfer limitation separately.

Pesticides and pharmaceuticals pose an inferential problem different from that posed by chlorinated solvents. The dominant risk may arise not from persistence of the parent compound alone, but from partial transformation, redox-dependent pathway switching, sorption-retention trade-offs, or metabolite formation that changes acceptability even when overall removal appears favorable. Soil biostimulation and bioaugmentation studies, biomix and biobed systems, denitrification-linked removal of pharmaceuticals, and strain-resolved degradation experiments show that parent-compound decline is a weak stand-alone endpoint (Banzhaf et al., 2012; Tortella et al., 2012; Ruíz-Hidalgo et al., 2016; Karas et al., 2016; Raffa and Chiampo, 2021; Triozzi et al., 2023; Xie et al., 2024). Recent bioaugmentation studies targeting nonsteroidal anti-inflammatory drugs (NSAIDs) with newly isolated bacterial strains strengthen this point: strain-level degradation potential is useful for intervention design, but decision-grade interpretation still requires metabolite tracking, endpoint toxicity, persistence of the introduced function, and evidence that degradation proceeds under field-relevant soil conditions (Cycoń et al., 2025; Klim et al., 2025; Klim et al., 2026). For these compounds, the twin must be metabolite-aware and redox-aware: it must ask whether the site is moving toward mineralization, detoxification, or only conversion to a different exposure problem. First-order approximations may remain admissible for bounded screening, but they should not be used as evidence of pathway completion, source depletion, or closure readiness without independent mechanistic constraints.

PFASs require separate decision logic because lower aqueous concentrations may result from retention rather than destruction. At aqueous film-forming foam-impacted sites, PFAS distributions can reflect hydrophobic partitioning, electrostatic interactions, air-water interfacial retention, salinity effects, matrix diffusion, and precursor transformation, so concentration attenuation cannot be interpreted as biodegradation without a mass and transport context (Adamson et al., 2022; LaFond et al., 2023; Zhang et al., 2025; Nasrollahpour et al., 2026). Microbial evidence also requires stricter wording than for many hydrocarbon or chlorinated-solvent systems. Enzyme potential, gene presence, or loss of a precursor signal supports a biotransformation hypothesis, not a destruction claim, unless product profiles, fluorine release, and mass balance are consistent with cleavage of carbon-fluorine bonds and durable reduction of hazard (Hu and Scott, 2024). For PFAS-impacted sites, a decision-grade twin should state which claim is being tested, i.e., precursor conversion, mobility reduction, retention, partial defluorination, toxicity reduction, or full mineralization. The minimum evidence panel should include precursor and product profiles, adsorbed and dissolved mass, retention diagnostics at air-water and solid-water interfaces, fluoride release where mechanistically justified, total fluorine or extractable organofluorine balance where feasible, product mobility, endpoint toxicity, total mass control, and reporting limits. Without that panel, a twin risks converting analytical non-detection or phase transfer into false assurance.

Biotechnology-assisted remediation adds another decision layer because biological or amendment-based enhancement can improve removal while also changing exposure, crop uptake, soil function, or endpoint acceptability. Plant-microbe systems illustrate this problem. The symbiotic association between *Sinorhizobium* sp. W16 and soybean increased fomesafen degradation in soil and reduced herbicide stress on nitrogen fixation, whereas corn-bacteria combinations have been reported to increase pyrene removal while also affecting crop biomass and contaminant accumulation in plant tissues (Chen et al., 2023; Gao et al., 2025). For pesticide-contaminated agricultural soils, imidacloprid uptake by vegetables shows why parent-compound dissipation cannot be separated from plant accumulation and food-chain relevance (Li et al., 2019). Amendment-based approaches create a related distinction: ball-milling-modified biochar can passivate Cd and reduce crop uptake, but such an intervention supports a mobility-control claim rather than a biodegradation claim (Lu et al., 2025). Other biotechnology-oriented strategies, including triacontanol priming under chromium stress, microalgal strain adaptation and microbiome optimization, and saline-soil amelioration research, further show that remediation endpoints may include stress tolerance, productivity, soil function, and land-use recovery rather than contaminant destruction alone (Zhang et al., 2024; Ahmed et al., 2025; He et al., 2025). A decision-grade twin should therefore state whether the biotechnology claim concerns degradation, passivation, uptake reduction, crop safety, ecological function, or agronomic recovery.

These contaminant comparisons also expose a larger conceptual mistake. The literature often separates intervention design from evidence design, as though the former could be planned around a technology label and the latter added later as monitoring. That separation does not hold under field conditions. Oxygen delivery, donor dosing, recirculation, bioaugmentation, and controlled-release amendments are not just technologies. They are perturbations intended to test whether a particular microbial limitation is actually controlling the site. When framed this way, the value of a digital twin becomes more precise. It is not there to digitize operations in the abstract. It is there to determine whether the intended microbial process was engaged, whether the controlling limitation shifted, and whether the system has entered a state in which intensification no longer changes the outcome. This is why flux-based metrics, end-product formation, functional biomarkers, and redox-regime diagnostics are often more informative than additional concentration mapping. They reveal mechanism-level success or failure, not only plume behavior.

The critical judgment from this section is direct. There is no transferable control logic for *in situ* bioremediation that is independent of contaminant class, pathway structure, and the dominant source of inferential error. Hydrocarbon systems are frequently limited by accessibility, chlorinated solvents by pathway completion under competitive redox chemistry, pesticides and pharmaceuticals by partial transformation and metabolite risk, and PFASs by the distinction between precursor transformation, mobility control, toxicity of products, and defluorination. A decision-grade twin must be built around these differences. Once that requirement is ignored, the digital layer becomes generic at the cost of scientific meaning.

## 4 Evidence-role matrix for causal attribution in field bioremediation

Evidence integration should begin with the ambiguity that must be reduced. Plausibility evidence asks whether a pathway could operate. Attribution evidence tests whether reaction, rather than transport or retention alone, explains the observation. Constraint evidence bounds model states, parameters, domains, or boundary conditions. Trigger evidence justifies action only when the tolerated uncertainty and alternative explanations are stated. Table 5 therefore separates evidence role from inference strength and from the capacity to defeat a transport-only explanation.

**TABLE 5:** Evidence roles, inference strength, and ability to defeat transport-only explanations.

Evidence stream	Primary role	Inference strength for biodegradation	Can defeat a transport-only hypothesis by itself?	Required coupling before action
Concentration trends and daughter products	Screening and pathway progress	Low to moderate	No	Flux, CSIA, mass balance, redox, or product stoichiometry
Geochemistry, electron acceptors, pH, alkalinity, ORP	Plausibility and regime diagnosis	Low alone, moderate when time-resolved	No	Contaminant-specific products, biomarkers, or isotope evidence
Mass flux, mass discharge, and geophysics	Transport and delivery constraint	Moderate for transport state, low for reaction mechanism	No	Chemistry, isotope, or biological evidence
Functional genes and qPCR	Biological capacity	Low to moderate	No	Transcripts, products, redox compatibility, and transport context
Transcripts and activity-linked biomarkers	Capacity-activity separation	Moderate	No	Product formation, geochemistry, and spatial co-location
CSIA	Attribution of reaction *versus* transport	High when fractionation and mixing limits are addressed	Often yes, for reaction occurrence, not for full pathway closure	Daughter products, mass balance, and CSM transport constraints
Toxicity and bioeffect endpoints	Acceptability and closure veto	High for endpoint acceptability, low for attribution	No	Chemical identity, products, and exposure pathway analysis
Multi-line field campaign	Causal convergence or conflict	High when lines are independent	Yes, if independent lines reject dilution, retention, and redistribution	Explicit CSM alternatives and uncertainty statement

CSIA, compound-specific isotope analysis; CSM, conceptual site model; ORP, oxidation-reduction potential; qPCR, quantitative PCR. Inference strength refers to the ability of an evidence stream to support a biodegradation or transformation claim under field conditions. “By itself” means without independent coupling to transport, geochemistry, products, biomarkers, isotope data, mass balance, or toxicity endpoints. Even high-strength evidence may be insufficient for closure if pathway completion, rebound risk, or residual acceptability has not been demonstrated.

The matrix should be read as a sequencing tool. Geochemical indicators are strongest when they exclude impossible pathways and define the admissible process space. They are weaker when used to infer pathway completion or contaminant-specific destruction, because those claims remain vulnerable to spatial heterogeneity, temporal instability, and hydraulic redistribution (Kao et al., 2008; Song et al., 2017; Ma et al., 2020; Sale et al., 2021; Davis et al., 2023; Romantschuk et al., 2023). Molecular and isotopic evidence matter most when the field question is causal attribution rather than plausibility alone. Molecular monitoring should therefore distinguish genetic potential, in situ activity, and verified pathway operation, because gene detection, transcript abundance, product formation, and reaction flux do not carry the same inferential weight (Cycoń, 2026). Functional genes, transcripts, and CSIA can then constrain pathway progress and reaction-versus-transport ambiguity, but only when interpreted inside a transport-aware CSM (Rahm and Richardson, 2008; Scheutz et al., 2008; Blessing et al., 2009; Hunkeler et al., 2011; Ottosen et al., 2021; de Vera et al., 2022; Cycoń, 2026). Geophysical evidence deserves separate caution because electrical and petrophysical responses are non-unique, yet field-scale resistivity and imaging can improve interpretation of reactive-zone geometry and delivery performance when constrained by chemistry and site structure (Flores Orozco et al., 2011; Sentenac et al., 2015; Nivorlis et al., 2024). Figure 2 converts this static evidence-role matrix into a process map. It complements Table 5 by showing interference flow rather than classifying evidence strength, and it shows how evidence streams move from plausibility testing to attribution, model constraint, and field judgment.

Real-time sensing and spatial monitoring form a separate category because they answer a different question. i.e., where and when the site departs from the static assumptions under which most remediation interpretations are made. Continuous ORP monitoring, passive flux measurements, and geophysical imaging do not, by themselves, prove biodegradation. What they do is often more important. They reveal regime switching, hydraulic instability, reactive-zone displacement, and mass-transfer constraints that would otherwise invalidate mechanistic interpretation built from sparse sampling. Field studies that combine high-frequency process sensing with flux or spatial data show the same pattern across different technologies: temporal density is useful only when it reduces state ambiguity that matters for action, and spatial density is useful only when it resolves the geometry of transport and treatment rather than multiplying redundant measurements (Verreydt et al., 2013; Refsgaard et al., 2016; Horst et al., 2022; Huang et al., 2022; Davis et al., 2023; Nivorlis et al., 2024). These evidence streams are therefore decision-relevant not because they are modern, but because they can expose when a static interpretation has already failed.

A final category, too often treated as optional, concerns bioeffect and ecotoxicity endpoints. Chemical improvement does not necessarily mean biological acceptability. Studies on PAH treatment and amendment-driven side effects have shown that residual toxicity may persist or even worsen when additives or intermediate products alter the exposure profile, despite favorable removal statistics. In contaminated soil and groundwater, this matters because intervention success is judged not only by disappearance of the target analyte, but by whether the remaining system is environmentally acceptable under the intended land use and exposure context (Hamdi et al., 2007; Frutos et al., 2010; Tillotson and Borden, 2015). Any evidence-role matrix that ignores this endpoint risks confusing process performance with remediation success. The consequence is procedural. Evidence streams should be added only when they reduce a named ambiguity: feasibility, attribution, model constraint, trigger sufficiency, or acceptability. Once that role is stated, evidence integration stops functioning as a rhetorical claim and becomes a field test of which causal explanations remain defensible.

## 5 What models can infer, and what they cannot

The modeling literature in contaminated-site remediation often asks the wrong question. It asks which model class is most advanced, fastest, or easiest to deploy, when the relevant question is narrower. i.e., what decision claim can a given model defend under the evidence constraints of a field site. This distinction matters because the same data record can support very different levels of inference. A model that reproduces concentration trajectories may still fail to distinguish biodegradation from dilution, miss the process controlling late-stage persistence, or fit incompatible site hypotheses with comparable error. In decision-grade work, model admissibility should be judged by the claim it is asked to support, not by formal sophistication or computational cost (Refsgaard et al., 2016; Song et al., 2017; Haasnoot et al., 2018; Davis et al., 2023; Verhaeghe et al., 2024; Shah et al., 2026). For chlorinated ethenes, recent review work reinforces this point: RTMs are most useful when they encode pathway structure, transport, and field constraints together, not when they merely reproduce concentration histories (Luo et al., 2024). Table 6 specifies the claim and validation gate for each model class, while Table 7 separates interpretive hazards and defensible uses.

**TABLE 6:** Model classes, admissible claims, and minimum validation gates.

Model class	Admissible decision claim	Minimum evidence burden	Minimum validation gate	Representative references
RTMs	Whether observed change is more consistent with reaction, transport, or both	Hydrogeology, boundaries, concentration time series, and at least one independent mechanistic constraint	Flow and transport validation outside reaction calibration; residual checks by location and time; mass-transfer scenario	Chambon et al. (2013); Viotti et al. (2014); Höyng et al. (2015); Davis et al. (2023); Luo et al. (2024)
Pathway and kinetic process models	Whether transformation can proceed to acceptable end products under site conditions	Parent and daughter products, redox context, biomarkers or CSIA	Parent-product consistency; electron-balance check; sensitivity to competing pathways	Hunkeler et al. (2011); Chambon et al. (2013); Ottosen et al. (2021); De Vera et al. (2022)
Hybrid process-data models	Whether short-horizon predictions improve operations without losing process accountability	Process inputs, routine chemistry, QA/QC-controlled data streams, and stated update logic	Temporal holdout; drift test; documented split between process and learned terms	Aliashrafi et al. (2021); Schneider et al. (2022); Dihan et al. (2024); Verhaeghe et al. (2024)
Surrogates and emulators	Whether bounded scenario screening can be accelerated	Parent-model outputs or dense observations over a stated domain	Validation against parent-model runs outside training subset but inside the stated domain	Rasheed et al. (2020); Tran et al. (2021); Davis et al. (2023)
Data-driven surveillance models	Whether anomalies or short-horizon deviations can be flagged	High-frequency, quality-controlled, time-resolved data with stable semantics	Temporal holdout; sensor-drift test; no closure or pathway-completion claim without independent LoE	Dogo et al. (2019); Chen et al. (2020); Huang et al. (2022); Davis et al. (2023)
Functional microbial models	Whether biological capacity and activity are sufficient for a targeted process	qPCR, transcripts, products, geochemistry, and transport setting	Spatial and temporal match among biomarkers, products, redox state, and delivery	Rahm and Richardson (2008); Majone et al. (2015); Davis et al. (2023); Hu and Scott (2024); Cycoń (2026)
Community and genome-scale models	Whether observed shifts are plausible under energetic or competitive constraints	Genomic information, constraint data, and simplified field context	Field parameterization check; no direct field-rate or closure prediction without field evidence	Davis et al. (2023)

CSIA, compound-specific isotope analysis; LoE, line of evidence; QA, quality assurance; QC, quality control; qPCR, quantitative PCR; RTMs, reactive transport models. Validation gates define the minimum test required before a model output can support the stated decision claim. They do not imply that a model class is generally sufficient for closure, remedy switching, or regulatory acceptance without site-specific evidence and uncertainty bounds.

**TABLE 7:** Model hazards and defensible uses.

Model class	Dominant interpretive hazard	Do not trust it when	Most defensible use
RTMs	Structural error hidden by calibration	Low-permeability exchange, redox switching, or pathway structure is omitted	Separating reaction from transport and testing rebound or tailing
Pathway and kinetic process models	Equifinality among kinetic forms and incomplete pathway representation	Pathway closure is inferred from parent loss alone	Diagnosing ERD bottlenecks and donor competition
Hybrid process-data models	Learned residuals absorb missing processes	The learned component cannot be explained against site processes	Operational forecasting with an explicit process core
Surrogates and emulators	Extrapolation outside training domain	Regime change or a new intervention moves the site outside training bounds	Fast sensitivity ranking and bounded scenario screening
Data-driven surveillance models	Spurious correlation and drift sensitivity	Used to infer biodegradation, source depletion, or closure readiness	Anomaly detection and event recognition
Functional microbial models	Capacity-activity mismatch	Biomarkers are detached from redox, products, and transport	Bioaugmentation, donor revision, and stalled-function diagnosis
Community and genome-scale models	Laboratory assumptions are overextended to field rates	Treated as direct predictors of field-scale closure	CSM refinement and selection of simplified functional indicators

CSM, conceptual site model; ERD, enhanced reductive dechlorination; RTMs, reactive transport models. The hazards listed here identify conditions under which a model class can create misleading decision confidence. They should be read together with the validation gates in Table 6; a model may be useful for interpretation or screening without being admissible for closure, remedy switching, or control-linked action.

Hybrid and data-driven approaches require stricter interpretation than their operational convenience suggests. In field settings where iterative control, fast scenario screening, or repeated updates are needed, full physics-based simulation may be too slow or too underconstrained to serve operations directly. Hybrid architectures and emulators can help, but only if the process core remains identifiable and the learned component is prevented from silently absorbing structural misspecification. Work in water and process systems, along with recent remediation-oriented studies, shows that hybrids become scientifically admissible only when notation, calibration discipline, and model-to-data interfaces are explicit, and when the parent process structure remains the reference frame for interpretation (Corominas et al., 2010; Rasheed et al., 2020; Aliashrafi et al., 2021; Tran et al., 2021; Schneider et al., 2022; Verhaeghe et al., 2024). Pure data-driven models occupy a narrower domain. They can support anomaly detection, event classification, and short-horizon surveillance, but they should not infer remediation success, closure readiness, or pathway completion without an independent process interpretation (Dogo et al., 2019; Chen et al., 2020; Sale et al., 2021; Horst et al., 2022; Huang et al., 2022).

Microbial process models are indispensable where the central decision concerns biological function rather than only plume behavior. This is clearest in ERD, where donor choice, bioaugmentation, and reinjection decisions depend on whether the site can sustain dechlorination to ethene rather than merely reduce parent concentrations. It is equally relevant for pesticides, pharmaceuticals, and PFASs, where partial transformation, incomplete pathway closure, or incomplete defluorination may alter hazard more than parent removal suggests. Functional biomarkers, transcripts, pathway products, and competition-aware community models can improve interpretation when the decision turns on capacity versus activity, delayed function, or metabolite formation under changing redox states (Rahm and Richardson, 2008; Qureshi et al., 2009; Tortella et al., 2012; Chambon et al., 2013; Cycoń et al., 2017; Cunningham et al., 2020; Markowicz et al., 2021; Cycoń, 2026). In pharmaceutical-contaminated soils, strain-level bioaugmentation studies can support the feasibility of targeted degradation, but they should not be read as field-scale decision evidence unless degradation, persistence of function, metabolite behavior, and soil-context constraints are reported together (Klim et al., 2025; Klim et al., 2026). They do not become field-predictive merely because they are biologically explicit. Once detached from transport structure and amendment delivery, they become mechanistically interesting but operationally weak.

No model class is inherently decision-grade. A model becomes decision-grade only when its inferential burden matches the evidence available and the decision claim being made. RTMs are strongest for reaction-versus-transport discrimination. Hybrid and surrogate models are admissible for bounded operational forecasting if learned terms remain auditable. Purely data-driven systems belong to surveillance, not causal attribution. Microbial models are decisive when the field question is biological capability, pathway closure, or metabolite risk, but only when interpreted within their geochemical and transport context.

## 6 Uncertainty is not a residual problem; it is the engineering problem

Section 5 defines what each model class may claim. The next question is when that claim becomes inadmissible because uncertainty is too large, too structural, or too poorly observed. In subsurface bioremediation, uncertainty is not a residual term after monitoring and modeling. It is a design variable that determines which measurements have value, which forecasts can alter action, and which claims must remain conditional (Refsgaard et al., 2016; Song et al., 2017; Haasnoot et al., 2018; Davis et al., 2023; Verhaeghe et al., 2024). Table 8 links uncertainty classes to the specific forms of false confidence they generate and to the decision status they should impose. It should therefore be read as the rejection screen for Table 6, ie A model may be appropriate in class, but its decision claim fails if the relevant uncertainty class has not been diagnosed and bounded. Calibration is admissible only when the observation-to-state map prevents one data stream from compensating for the absence of another. Transport parameters, degradation kinetics, redox-state transitions, and pathway activity are not constrained by the same observations. Kinetics tuned without isotope, product, biomarker, or flux constraints can create numerical agreement with weak causal value. High-frequency data can intensify the same error when state and parameter updates occur without explicit QA/QC and update gates (Hunkeler et al., 2011; Adamson et al., 2016; Refsgaard et al., 2016; Wanner et al., 2016; Sale et al., 2021; Davis et al., 2023; Verhaeghe et al., 2024).

**TABLE 8:** Uncertainty classes, diagnostics, and decision status in decision-grade digital twins.

Uncertainty class	Typical manifestation in field bioremediation	Minimal diagnostic	Minimal mitigation action	False confidence created if ignored	Decision consequence	Decision status	References
Measurement and data-integrity uncertainty	Sensor drift, timestamp inconsistency, non-comparable laboratory outputs	QA/QC review, metadata completeness, cross-sensor checks	Data QA/QC pipeline, flagging and exclusion rules, provenance logging	Artificial regime shifts, false trigger events, biased updates	Incorrect dosing, switching, or escalation	Stop or hold if QA/QC fails; proceed only after flagged data are excluded or corrected	Horst et al. (2022); Huang et al. (2022); Davis et al. (2023); Molin et al. (2024)
Spatial heterogeneity	Strong concentration and redox gradients; point samples that do not represent the treated domain	Co-registered spatial LoE, near-well comparison, domain mapping	Add spatial evidence, relocate points, treat site as multi-domain	Apparent control based on one hydraulically favorable window	Overconfident interpretation of treatment reach and source depletion	Collect additional evidence or proceed with a domain-specific conservative envelope	Song et al. (2017); Davis et al. (2023); Nivorlis et al. (2024)|
Temporal variability and regime switching	Redox cycling, transient donor effects, episodic hydraulic disturbance	High-frequency process sensing where justified	Sequential state updating, regime-tied trigger logic	Stable process assumptions applied to unstable systems	Overreaction to noise or missed timing of interventions	Proceed only with state-specific trigger logic; otherwise collect additional evidence	Haasnoot et al. (2018); Sale et al. (2021); Davis et al. (2023)
Transport and mass-transfer limitation	Tailing, rebound, long persistence despite strong intervention	Stalled response under strong levers, low-permeability exchange evidence	Dual-domain or explicit mass-transfer representation	Apparent treatment failure misread as biological limitation, or apparent progress misread as depletion	Premature closure or futile intensification	Proceed with conservative rebound envelope; do not use for closure without mass-transfer constraints	Adams et al. (2011); Adamson et al. (2016); Wanner et al. (2016); Davis et al. (2023)
Parameter uncertainty	Wide plausible parameter ranges with similar fit quality	Sensitivity analysis against independent LoE	Parameter bounding with independent constraints	Best-fit narratives mistaken for settled system knowledge	Overstated confidence in schedules and remedy choice	Proceed with conservative bounds if the decision is insensitive; otherwise collect additional evidence	Refsgaard et al. (2016); Davis et al. (2023)
Practical non-identifiability	Multiple parameter sets or site hypotheses fit the same observations	Competing fits with similar residual error	Reduce parameter space, redesign monitoring using VoI logic	False precision in parameter values and forecasts	Misleading optimization and fragile operational planning	Collect additional evidence or redesign monitoring before optimization	Refsgaard et al. (2016); Haasnoot et al. (2018); Davis et al. (2023)
Structural uncertainty	Omitted pathways, wrong process form, misassigned domain exchange	Persistent prediction bias, contradiction among LoE	Test alternative structures, document exclusions explicitly	Good fit mistaken for mechanistic correctness	Strategy errors that no recalibration can repair	Stop or redesign until alternative process structures have been tested	Song et al. (2017); Davis et al. (2023); Verhaeghe et al. (2024)
Decision uncertainty and trade-offs	Similar predicted outcomes under different actions with different side effects	Scenario comparison under explicit objectives and constraints	Bounded scenarios, stated objectives, switch criteria	Apparent equivalence among options with different risk profiles	Unstable or unjustified strategy changes	Proceed only after objectives, constraints, and acceptable collateral effects are stated	Refsgaard et al. (2016); Song et al. (2017); Davis et al. (2023)

LoE, line of evidence; QA, quality assurance; QC, quality control; VoI, value of information.

**FIGURE 4:**
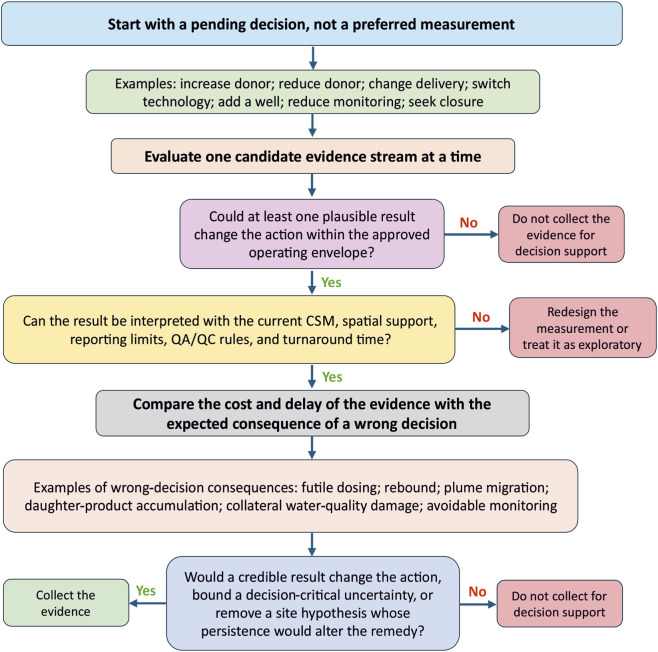
Decision tree for Value of Information in a remediation twin. The figure shows how a candidate evidence stream should be screened before it is added to a decision-grade monitoring program. The sequence begins with a pending management decision rather than a preferred measurement and evaluates whether a plausible result could change the action, whether the result is interpretable under the current site and data-quality constraints, and whether the expected decision value justifies the cost and delay of the measurement. It therefore operationalizes Value of Information as a decision-first procedure for evidence selection in remediation digital twins. CSM, conceptual site model; QA/QC, quality assurance and quality control.

Identifiability and structural uncertainty should be treated as stop conditions, not as technical caveats. Comparable residual errors may arise from different combinations of transport, reaction, source persistence, and low-permeability exchange. When competing structures remain viable, the correct output is not a single optimized remedy but a bounded decision: collect the evidence that can separate the structures, proceed only with conservative triggers, or narrow the claim to surveillance rather than attribution (Adams et al., 2011; Adamson et al., 2016; Wanner et al., 2016; Song et al., 2017; Aliashrafi et al., 2021; Schneider et al., 2022; de Vera et al., 2022; Davis et al., 2023; Verhaeghe et al., 2024). This is why value of information (VoI) should govern monitoring design. A measurement is worth taking only if it can change a decision, bound a critical uncertainty, or eliminate a site hypothesis that would otherwise remain viable. The point has deep practical implications. In ERD, end products and pathway-linked functional markers may be more valuable than additional precursor concentrations. In mass-transfer-limited systems, flux measurements and evidence of low-permeability exchange may matter more than denser concentration mapping. In redox-controlled treatment, high-frequency process sensing is worth the effort only if there is an actual lever to adjust and a predefined threshold that would justify adjustment (Rahm and Richardson, 2008; Scheutz et al., 2008; Verreydt et al., 2013; Refsgaard et al., 2016; Haasnoot et al., 2018; Huang et al., 2022; Davis et al., 2023). Once VoI is taken seriously, monitoring ceases to be a checklist exercise and becomes part of decision design. A decision-grade system is therefore judged by the uncertainty it can expose, reduce, and preserve in the decision record, not by the apparent precision of its preferred forecast. A practical difficulty in this VoI loop is the assignment of prior probabilities to competing CSMs. These priors should not be treated as subjective confidence in a preferred site story. They should be elicited as transparent weights based on source history, release timing, hydrostratigraphy, hydraulic gradients, concentration and daughter-product patterns, geochemical compatibility, amendment delivery records, rebound behavior, and known failure modes for the contaminant class. A useful field heuristic is to begin with equal weights for all plausible CSMs, then down-weight hypotheses that contradict independent lines of evidence and up-weight hypotheses that explain observations across scale, time, and mechanism without special pleading. Priors should remain coarse, for example, high, medium, low, or approximate probability bands, until the evidence justifies numerical refinement. When two hypotheses remain close in weight and imply different actions, the next measurement has high potential value only if it can separate them before the decision window closes. Figure 4 translates this VoI logic into a practical screening sequence, showing that a candidate evidence stream should be collected only when a plausible result could change the action, reduce decision-critical uncertainty, or eliminate a site hypothesis whose persistence would alter the remedy. Decision-grade status should not be treated as a universal deliverable. The standard is expensive because it requires observations that are independent enough to separate transport, reaction, delivery, redox state, and secondary effects. In low-risk or weakly controllable settings, a lower maturity target may be scientifically preferable: a traceable monitoring twin that preserves uncertainty and blocks overclaiming. The higher standard is justified when the measurement program can alter an active decision with material consequences for dose, delivery, shutdown, technology switching, monitoring reduction, or regulatory closure.

## 7 Decision logic, switching criteria, and secondary risks

The decisive failure of many remediation digital twins is not weak analytics, but weak judgment design. A system may integrate chemistry, biomarkers, flux data, and forecasts, yet still fail at the point where evidence must be converted into a field action. The underlying problem is conceptual. Remediation decisions are still too often treated as informal expert interpretation guided by concentration decline, rather than as explicit conditional rules tied to mechanism, uncertainty, and acceptable collateral effects. In that setting, digital twins do not discipline decisions. They merely decorate them. A decision-grade system begins only when the site team, the model, and the decision record state the same operational logic, i.e., what outcome matters, which observation can trigger a change, what alternative explanation must be ruled out, and what level of uncertainty is still tolerable before escalation, de-intensification, or closure is considered. This is the threshold most published systems do not cross.

The first consequence is that decision objectives must be stated in terms of risk and control, not only treatment response. The literature is consistent on this point across different contaminant classes and operational settings. Flux-based measurements in groundwater plumes show that migration control and exposure reduction can diverge from concentration maps, particularly where hydraulic variability or incomplete source depletion distort apparent plume improvement. Work on passive flux meters, barrier performance, risk-linked monitoring, and treated-effluent discharge zones indicates that mass discharge often carries more immediate decision value than point concentration because it relates directly to loading across a control plane and to the acceptability of management endpoints (Verreydt et al., 2013; Ma et al., 2020; Pishgar et al., 2022; Davis et al., 2023). The same logic applies in vadose-zone and source-zone settings, where high removal percentages can coexist with residual toxicity, mobilized intermediates, or persistent inaccessible mass (Frutos et al., 2010; Tillotson and Borden, 2015; Simpanen et al., 2016; Song et al., 2017).

A second consequence is that switching criteria must be evidence-anchored and mechanism-specific. In chlorinated solvent systems, the strongest triggers are not generic trend changes, but process-linked signs that the intended pathway either has or has not engaged. Field and laboratory evidence around enhanced reductive dechlorination shows that donor addition, bioaugmentation, end-product formation, and pathway-linked reductive dehalogenase biomarkers such as *vcrA* and *tceA* must be interpreted together. Ethene formation without sustained functional evidence may indicate unstable progression. Gene presence without end-product formation may indicate capacity without effective control. Apparent parent decline without pathway closure may conceal intermediate accumulation or donor competition (Rahm and Richardson, 2008; Scheutz et al., 2008; Hunkeler et al., 2011; Majone et al., 2015; Ottosen et al., 2021). Comparable caution emerges from air-sparging studies, where increasing air flux shortens time to early response but does not remove the late-stage tail governed by mass transfer (Adams et al., 2011; Adamson et al., 2016; O’Connor et al., 2018). The correct synthesis is narrow. A trigger should not be tied to “lack of improvement” in the abstract. It should be tied to evidence that a named controlling process has shifted or failed to engage.

This is where decision logic becomes qualitatively different from monitoring logic. Monitoring asks whether the site changed. Decision logic asks whether the interpretation of that change is strong enough to justify a new action. Continuous ORP measurements illustrate the difference well. High-frequency ORP can reveal regime switching that periodic sampling would miss, which is valuable where redox control is the intended intervention lever. Yet ORP should not be allowed to trigger action on its own. It must be read together with geochemical context, amendment history, and, where relevant, pathway-linked biological or product evidence. Otherwise, the site is reacting to a proxy for process state without testing whether that state matters for remediation performance (Sale et al., 2021; Horst et al., 2022; Huang et al., 2022; Davis et al., 2023). What follows from this comparison is straightforward. Triggers should be built around causal sufficiency, not around signal availability. Three examples illustrate the difference. In ERD, the twin should place donor escalation on hold when ORP and electron-acceptor depletion indicate reducing conditions but daughter-product progression, ethene formation, and vcrA or tceA activity do not improve across two update intervals. The default secondary-risk screen should also hold escalation when dissolved methane exceeds 5 mg/L or increases by more than 2 mg/L between consecutive rounds, dissolved sulfide exceeds 0.5 mg/L as S, pH falls below 6.0, alkalinity is being depleted relative to baseline, or arsenic, dissolved iron, or dissolved manganese crosses site-specific action criteria. Donor dosing should be de-intensified, not merely held, when methane exceeds 10 mg/L and continues to rise, sulfide exceeds 1.0 mg/L as S, headspace methane at any monitoring point exceeds 10% of the lower explosive limit, headspace hydrogen sulfide exceeds the approved site health-and-safety action level, or daughter products accumulate while terminal products remain stagnant. In bioventing, air delivery should be increased only when oxygen penetration is insufficient and respiration response indicates biological demand, while volatilization loss and inaccessible residual mass have been separated from biodegradation. In controlled-release donor systems, reinjection or formulation change should be triggered by evidence of poor donor distribution, short residence time, or failure to sustain the intended redox window, not by a parent-compound plateau alone.

The most neglected part of decision logic concerns secondary risks. Here the field repeatedly understates what it already knows. Several LoE show that remediation can look chemically successful while becoming less acceptable environmentally. Residual toxicity can persist despite substantial contaminant removal, especially when additives or transformation products change the biological effect profile of treated soil or groundwater (Hamdi et al., 2007; Frutos et al., 2010; Tillotson and Borden, 2015). In reductive systems, donor loading can degrade water quality, alter gas production, or mobilize constituents that were not previously the main management concern (Tillotson and Borden, 2015; Song et al., 2017; Davis et al., 2023). Intensive stimulation may also create biological side effects, including shifts in antibiotic resistance markers, which do not always belong in the primary endpoint but cannot be excluded from a serious judgment about intervention acceptability (Cunningham et al., 2020; Markowicz et al., 2021; Cycoń et al., 2019; Davis et al., 2023). The proper conclusion is not that every twin must model every collateral effect. It is that no twin deserves decision-grade status if it treats secondary impacts as *post hoc* commentary rather than as admissibility constraints on action. The secondary-risk panel should be selected by decision context rather than copied as a fixed analyte list. Five questions determine the minimum panel, i.e., Is the intervention reducing, oxidizing, or bioaugmenting? Is PFAS mobility or precursor transformation part of the claim? Are redox-mobile metals present or plausible? Is closure or monitoring reduction being considered? Would a secondary effect change regulatory acceptability? Table 9 specifies the resulting selection logic. This is particularly relevant for pharmaceutical bioaugmentation, where recent NSAID-degradation studies show the value of strain selection while also reinforcing the need to track transformation products, toxicity, and persistence of the introduced function before inferring treatment sufficiency (Cycoń et al., 2025; Klim et al., 2025; Klim et al., 2026).

**TABLE 9:** Selection algorithm for secondary-risk indicators.

Decision context	Required indicators	Conditional indicators	Stop or hold condition
Reducing system, including ERD	Methane, dissolved and headspace where gas exposure is plausible; sulfide as S, dissolved and headspace hydrogen sulfide where gas exposure is plausible; dissolved fe; dissolved Mn; arsenic where redox mobilization is plausible; TOC or COD; residual donor; pH; alkalinity; sulfate; nitrate; daughter products; ethene or other terminal products	Soil-gas or vapor monitoring near receptors; gas-flow or odor logs; sulfide precipitation indicators; ferrous iron amendment record where used; health-and-safety meter data; methane receptor survey; aquifer zones with high sulfate and low reactive iron	Hold automatic donor escalation at methane >5 mg/L, methane increase >2 mg/L per update interval, sulfide >0.5 mg/L as S, pH < 6.0, alkalinity depletion relative to baseline, metals above site criteria, or stalled daughter-product progression across two update intervals. De-intensify donor dosing or revise delivery at methane >10 mg/L and rising, sulfide >1.0 mg/L as S, headspace methane >10% LEL, approved hydrogen sulfide action level exceeded, redox-mobile metal mobilization, or daughter-product accumulation without terminal-product progress
Oxidative or aerated system	Oxygen demand, partial-oxidation products, rebound after stimulation is reduced or stopped, residual toxicity	Mobilized metals, pH shift, volatilization indicators	Apparent removal with increased toxicity, mobilization, or rebound
Bioaugmentation	Introduced function or target-gene abundance, expression where relevant, daughter-product or metabolite progression, persistence of degradation phenotype, donor and redox compatibility	Strain persistence, broader microbiome shifts, antibiotic-resistance markers, metabolite-specific toxicity, or NSAID-specific transformation products when amendment strategy, pharmaceutical class, or receptor sensitivity warrants it	Gene presence without functional progression, or persistence of introduced function without acceptable product profile
PFAS-related claim	Precursor and product profile, adsorbed and dissolved mass, mobility indicators, reporting limits	Fluoride release, total fluorine or organofluorine balance, toxicity of products, air-water interfacial retention indicators	Lower dissolved PFAS concentrations without mass accountability
Closure or monitoring reduction	Rebound indicators, mass flux or discharge where relevant, toxicity or bioeffect endpoint, uncertainty statement	Additional indicators required by site-specific receptors or regulatory endpoints	Any unresolved secondary impact that could change acceptability

COD, chemical oxygen demand; ERD, enhanced reductive dechlorination; NSAIDs, nonsteroidal anti-inflammatory drugs; PFAS, per- and polyfluoroalkyl substances; TOC, total organic carbon. The panel should be selected according to intervention type, site geochemistry, regulatory decision, and plausible collateral effects. Required indicators are the minimum set for the listed decision context. Conditional indicators should be added when site history, amendment chemistry, receptor sensitivity, or regulatory endpoints make the secondary effect decision-relevant. Numeric trigger values are default operational screens for ERD, not cleanup levels or universal toxicity criteria. More stringent permit, receptor, vapor-intrusion, or health-and-safety values override them. Dissolved methane above 10 mg/L should be treated as a gas-risk screening condition; headspace methane above 10% LEL, should stop donor escalation and initiate safety review before further donor addition. Sulfide thresholds are conservative field action levels because sulfide formation reflects sulfate reduction and can impair dechlorination, generate odor and corrosion issues, or shift health-and-safety risk. The thresholds should be tightened in high-sulfate, low-iron, confined-space, or sensitive-receptor settings (IDEM, technical guidance document, 2019; NAVFAC EXWC, 2015; NIOSH, 2013).

Apparent loss of treatment efficiency has two distinct meanings. Diminishing returns means that the intervention is working but no longer accesses the controlling mass fraction. Functional failure means that the intended pathway never stabilized, was displaced, or produced unacceptable intermediates. These diagnoses require different data and different actions (Table 10).

**TABLE 10:** Diagnostic distinction between diminishing returns and functional failure.

Field symptom	Likely diagnosis	Discriminating data	Decision
Early decline followed by plateau, with falling flux and stable end products	Diminishing returns	Flux or discharge trend, rebound test, mass-transfer scenario, stable pathway products	De-intensify, shift endpoint, or redesign for mass-transfer control
Parent decline with daughter-product accumulation and weak end-product formation	Functional failure	Parent-daughter stoichiometry, ethene or terminal products, redox, *vcrA* or *tceA*, CSIA where available	Revise donor, bioaugment, improve delivery, or switch technology
ORP reaches target range but products and biomarkers do not progress	Functional failure or donor competition	Electron acceptors, methane, sulfide, donor residual, biomarkers, products	Hold automatic dosing escalation until mechanism is confirmed
Concentrations rebound after shutdown or hydraulic change	Mass-transfer limitation, not necessarily biological failure	Rebound test, low-permeability evidence, flux, dual-domain scenario	Do not close; maintain conservative rebound envelope
Chemical removal with persistent or increased toxicity	Acceptability failure	Toxicity endpoint, product profile, pH, metals, amendment residuals	Block closure or revise treatment objective

CSIA, compound-specific isotope analysis; ORP, oxidation-reduction potential; *tceA*, trichloroethene reductive dehalogenase gene; *vcrA*, vinyl chloride reductive dehalogenase gene. Diminishing returns and functional failure should not be inferred from concentration trends alone. The distinction requires evidence that separates mass-transfer limitation, pathway instability, donor or electron-acceptor competition, rebound, and endpoint acceptability. The decision column identifies the appropriate management response after the dominant diagnosis has been supported by the listed discriminating data.

A remediation digital twin becomes field-relevant only when it encodes objectives as risk-linked endpoints, translates mechanism-specific evidence into explicit switching criteria, and treats secondary impacts and durability risks as first-order decision variables. Systems that predict site behavior without governing the logic of strategy revision may support interpretation, but they do not qualify as decision-grade instruments.

## 8 Reporting standards and operational governance

**FIGURE 5:**
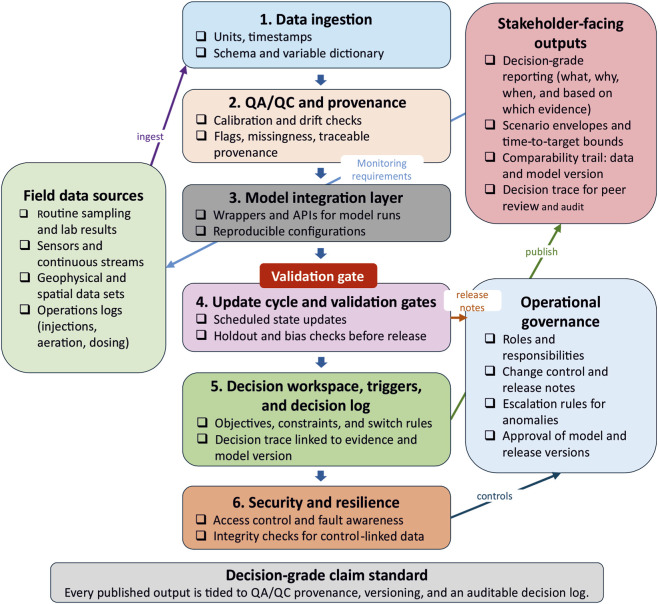
Operational workflow for audit-ready remediation twins. The workflow links field data, QA/QC, provenance, model updates, trigger rules, decision logs, and control interfaces. Security and rollback logic are required when the twin influences dosing, recirculation, aeration, alarms, or escalation. API, application programming interface; QA, quality assurance; QC, quality control.

The final weakness of the field is rarely discussed with enough precision. Even when site interpretation is conceptually careful, digital-twin claims often collapse at the level of deployment and reporting. The failure mode is familiar, i.e., unstable data streams, ambiguous variable definitions, undocumented preprocessing, opaque update cycles, weak provenance, and no decision trace showing why a strategy changed when it did. Under those conditions, the scientific question is no longer whether the remediation logic was sound. It is whether the claimed logic can be reconstructed and audited at all. In other words, governance is not a technical appendix to digital twins. It is part of the evidentiary burden. A system that cannot document how evidence moved into action cannot support a decision-grade claim, regardless of how sophisticated its modeling appears. Auditability should therefore be framed as a scientific validity condition: without a reconstructable chain from raw data to evidence state, model release, trigger, approval, and field action, the result is not a decision claim but an undocumented operational episode. Figure 5 locates deployment, governance, and decision trace within one operational workflow.

### 8.1 Minimum reporting standard for scientific claims

Scientific reporting should not restate the admission test. It should allow readers and regulators to reconstruct the workflow. A publishable decision-grade claim must therefore report the data dictionary, CSM ontology, data provenance, QA/QC rules, model-update schedule, validation gates, treatment of censored data, versioned decision log, model-release approvals, and control-linked safety logic. Work on natural source zone depletion, soil digital representations, model wrapping, and water-sector digitalization shows that this semantic and operational layer determines whether a digital-twin claim can be reproduced rather than merely described (Sookhak Lari et al., 2022; Silva et al., 2023; Jedermann and Lang, 2022; Zekri et al., 2022; Quaranta et al., 2023; Molin et al., 2024). Trigger documentation should also report the prespecified action threshold, uncertainty tolerance, and decision authority (Lund et al., 2018; Davis et al., 2023; Durden, 2025).

Approval should not be a generic sign-off. For a recommendation system, the twin may propose an action, but the responsible remediation professional authorizes the strategy shift after reviewing the evidence bundle, uncertainty state, and secondary-risk screen. For a control-linked system, automatic adjustment is allowed only inside a preapproved operating envelope defined by dose limits, flow limits, safety triggers, permitted monitoring points, and rollback conditions. Any action outside that envelope is a strategy change and requires named approval by the project manager or site owner, the responsible professional, and the regulatory contact where the permit, decision document, or closure pathway would be affected. The versioned decision log must record the rule fired, evidence bundle, data version, model version, uncertainty class, proposed action, authorized action, approver name and role, timestamp, operating-envelope status, permit status, and rollback requirement. Table 11 specifies the information that must be reported for that claim to be audited, reproduced, and linked to field action.

**TABLE 11:** Minimum reporting checklist for decision-grade remediation digital twins.

Reporting item	Required content	Editorial purpose
Variable dictionary	States, parameters, units, spatial support, timestamps, detection limits, censored-data rules	Prevents semantic ambiguity
CSM ontology	Source zones, pathways, domains, boundary assumptions, alternative hypotheses	Shows what the twin is allowed to infer
Data pipeline	Field acquisition, laboratory sources, QA/QC, preprocessing, exclusions, metadata	Makes input data auditable
Observation-to-state map	Which observation constrains which state, parameter, boundary, trigger, or exclusion test	Prevents evidence stacking
Model-update record	State updates, parameter updates, structural changes, release dates, approval status	Distinguishes learning from refitting
Validation record	Holdout checks, independent wells, independent LoE, intervention-event tests, residual pattern checks	Prevents fitted history from being reported as decision evidence
Uncertainty record	Main uncertainty classes, non-identifiable terms, scenario bounds, stop conditions	States what remains unresolved
Trigger and decision log	Predefined thresholds, action allowed, uncertainty tolerated, person approving, time of action	Links evidence to action
Secondary-risk record	Required panel, conditional additions, exceedances, response	Shows collateral effects were decision constraints
Regulatory crosswalk	Applicable permit or decision document; cleanup criteria; points of compliance; allowed operating envelope; notification triggers; change-control route; closure evidence package; regulator-visible exceptions	Prevents adaptive control from changing regulatory obligations without authorization
Security and resilience	Access control, sensor-fault handling, rollback, alarm behavior, recovery procedure	Protects control-linked decisions

CSM, conceptual site model; LoE, line of evidence; QA, quality assurance; QC, quality control. The checklist specifies reporting requirements, not additional admission criteria. Its purpose is to make the decision-grade claim auditable by documenting variable definitions, data provenance, model updating, validation, uncertainty, trigger logic, secondary-risk handling, and operational governance.

### 8.2 Operational governance for control-linked systems

Operational governance becomes a separate requirement when the twin affects field action. Systems that influence dosing, recirculation, aeration, amendment release, alarms, or escalation cannot be evaluated only on predictive merit. They must preserve integrity under data faults, missing streams, software errors, inappropriate access, and model-release mistakes. In remediation, the failure modes are concrete, i.e., an erroneous donor-dosing recommendation can drive methanogenesis, sulfide production, or arsenic mobilization; a false ORP alarm can trigger unnecessary amendment addition; delayed detection of redox-driven metal release can shift the risk profile before the contaminant endpoint improves; loss of sensor data during a hydraulic change can hide rebound or reactive-zone displacement. Governance requirements should therefore scale with control authority. A visualization-only system requires provenance and QA/QC. A recommendation system requires version control, approval records, and release notes. A control-linked system requires access control, fault detection, rollback logic, and documented responsibility for approvals (Tao and Qi, 2019; Jedermann and Lang, 2022; Molin et al., 2024; Durden, 2025; Underhill et al., 2026). A published claim that omits hypotheses, evidence roles, update logic, uncertainty bounds, trigger rules, provenance, and governance cannot be evaluated at the level it claims.

### 8.3 Regulatory interface for dynamic twins under static permits

Dynamic updating does not remove the legal force of the governing regulatory instrument, for example, a permit, record of decision, remedial action plan, consent order, or closure agreement. A decision-grade twin should therefore encode a regulatory envelope: approved amendments, maximum injection rate or dose, monitoring points, sampling frequency, action levels, points of compliance, reporting schedule, and conditions requiring regulator notification or permit modification. Within that envelope, the twin can recommend dose adjustment, pulse timing, recirculation change, aeration adjustment, or additional sampling when the governing document allows adaptive management. Outside it, the twin must stop at a recommendation, flag the proposed action as out-of-envelope, and route the decision to formal change control.

Closure determinations require an even stricter separation. The twin may assemble evidence for rebound control, flux reduction, product acceptability, pathway completion, and secondary-risk control, but it must not silently redefine cleanup levels, compliance points, exposure assumptions, or monitoring-duration requirements. The practical coexistence model is therefore a preapproved adaptive-management envelope: fixed regulatory endpoints, bounded operational adaptation, versioned evidence packages, regulator-visible exceptions, and a clear rule for when model learning becomes a permit-relevant change rather than routine implementation.

## 9 Conclusion and future perspectives

This review argues for a stricter standard for digital twins in contaminated soil and groundwater. The central question is not whether the site changed, whether a dashboard is current, or whether a model reproduces historical observations. The question is whether the reported system reduces ambiguity that can affect field action under heterogeneity. The admission test applied in Table 4 shows why many valuable studies remain evidence components, monitoring systems, model frameworks, or feasibility demonstrations rather than full decision-grade remediation twins. That distinction is not a criticism of those studies. It is a boundary on the claims that can be made from them.

The next advance will not come from calling more systems digital twins. It will come from making the label harder to earn. Studies should be designed around inferential closure rather than data availability, with pollutant-specific and pathway-specific control logic where partial transformation, metabolite risk, donor competition, or low-permeability mass exchange govern the outcome. Monitoring networks should follow value-of-information logic, but the required evidence density must be proportional to the decision at stake. Models should state what they can decide, what they cannot decide, and what alternative explanations remain after calibration. Reporting should expose the chain from observation to update to approval to action without gaps in provenance, version control, regulatory crosswalk, or decision trace.

A hypothetical ERD site illustrates how the framework is intended to operate. The initial CSM contains three plausible explanations for declining trichloroethene concentrations: productive reductive dechlorination, dilution or redistribution, and donor-responsive but incomplete dechlorination with daughter-product accumulation. The admission test first asks whether the decision is only operational, such as adjusting donor pulse timing, or higher-stakes, such as technology switching or closure. Table 5 then determines which evidence streams can separate the hypotheses: parent-daughter stoichiometry, ethene, redox state, CSIA where available, *vcrA* or *tceA* activity, mass flux, and evidence of delivery to the reactive zone. The contaminant-specific logic in Section 3 prevents the twin from treating low ORP or parent-compound decline as proof of pathway completion.

The decision action is then governed by trigger criteria rather than by trend interpretation alone. If daughter products stall, terminal products do not increase, functional markers remain weak, and methane or sulfide crosses the operational screens in Table 9, the twin should hold or de-intensify donor addition rather than escalate it. Table 10 then translates the field symptom into a diagnosis, for example, functional failure, donor competition, mass-transfer limitation, or secondary-risk onset, and routes the decision to an approved action with a versioned decision log. The final output is not a single claim that the site is improving. It is a bounded decision: continue, revise delivery, collect discriminating evidence, switch technology, or block closure until the unresolved hypothesis no longer controls the risk.

Future work should move from proof-of-concept digital twins to field tests that report how inference changed action. The highest priority is not another dashboard or calibrated concentration forecast, but comparative site studies in which independent evidence streams are added sequentially and their effect on donor dosing, aeration, amendment delivery, bioaugmentation, monitoring frequency, technology switching, or closure judgment is recorded. Such studies should report cases in which the twin changed the decision and cases in which it prevented action because attribution remained unresolved. Negative or conditional decisions are scientifically valuable when they show that a plausible CSM could not be rejected.

A second priority is the development of mechanism-specific trigger libraries. Chlorinated-solvent ERD, hydrocarbon bioventing, pesticide and pharmaceutical transformation, PFAS precursor conversion, and biotechnology-assisted soil remediation do not fail in the same way. Future studies should therefore report trigger performance by contaminant class and decision type, including false alarms, missed rebound, secondary-risk onset, daughter-product accumulation, bioeffect endpoints, crop uptake where relevant, and the conditions under which intensification no longer changes the controlling mass fraction. Biomarkers, transcripts, isotope signals, geophysical data, flux metrics, toxicity endpoints, and plant or crop endpoints should not be added as parallel descriptors. They should answer named decision questions about pathway activation, pathway failure, metabolite acceptability, exposure reduction, and land-use acceptability.

A third priority is governance and regulatory translation. Decision - grade twins will not be accepted for high - stakes use unless model updates, data versions, uncertainty states, trigger rules, approvals, rollback conditions, and regulatory - envelope status are preserved in an auditable record. Future reports should therefore include the decision log, not only the model result. They should also distinguish decisions made within an approved operating envelope from decisions that require formal change control. The next stage of the field should be judged by evidentiary discipline rather than by terminology: a decision - grade twin must state what causal claim is being made, which evidence can defeat it, which uncertainty remains, who approved the action, and why the action is justified.
